# Enhancing Thermogalvanic Efficiency through Electrostatic Interaction in Cationic Hydrogels

**DOI:** 10.1021/acsaem.4c02835

**Published:** 2025-01-08

**Authors:** Carlos M. Andreu, Ana López-Hazas, Sonia Merino, Ester Vázquez, Oscar J. Dura

**Affiliations:** † Instituto Regional de Investigación Científica Aplicada (IRICA), Universidad de Castilla-La Mancha, Ciudad Real E-13071, Spain; ‡ Facultad de Ciencias y Tecnologías Químicas, Universidad de Castilla-La Mancha, Ciudad Real E-13071, Spain; § Departamento de Física Aplicada, Universidad de Castilla-La Mancha, Ciudad Real E-13071, Spain

**Keywords:** energy harvesting, thermoelectrochemistry, thermogalvanic, hydrogels, 3D-Printing

## Abstract

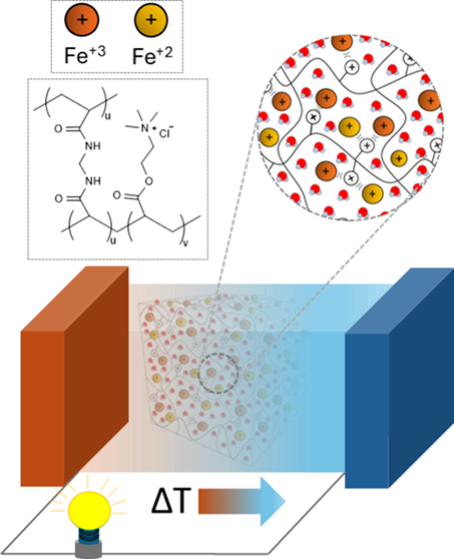

Thermoelectric hydrogels have the potential to be used in energy conversion devices for harnessing ubiquitous low-grade heat and generating useful electricity. This can be achieved through the use of thermogalvanic cells based on redox chemistry. While significant attention has been focused toward maximizing voltage for a given temperature gradient in liquid-based thermocells, it is crucial to consider both voltage and current density for accurate power output estimation in the case of gel-based thermocells. Here, we analyze the influence of the functional groups and the redox pair concentration over the voltage and current density in two different hydrogels. Our results confirm a path to enhance the current density in thermogalvanic hydrogels by incorporating a cationic pair into a cationic electroactive network (CN). This approach facilitates the movement of redox pairs, therefore increasing the power density output.

## 1 Introduction

The thermogalvanic conversion, which allows the reversible transformation between heat and the electrochemical potential,^1^ is nowadays attracting attention due to its great potential as waste heat recovery and refrigeration.^2−4^ This becomes particularly interesting at room temperature, where substantial heat is regularly wasted in several situations. In a thermogalvanic cell, also known as a thermocell, a solution containing both oxidation states of a redox pair comes into contact with two electrodes held at different temperatures. Therefore, this setup induces a potential difference (Δ*V*) through the electrodes, maintaining a temperature difference (Δ*T*) between them. This effect resembles the thermopower obtained in semiconductors via the common Seebeck effect.^5^ However, it is important to note that the source of the Δ*V* differs in thermocells compared with classical semiconductors. Whereas in the semiconductors, the potential difference is obtained from a charge carrier gradient, in thermocells, it results from the temperature dependence of the standard electrode potential and subsequently from the temperature dependence of the redox reaction.^5^ Thus, in the case of thermocells, the voltages obtained in typically redox pairs are quite greater (>1 mV/K) than those typically obtained in semiconductors (≈200 μV/K).^6^ The thermogalvanic conversion can be characterized by the “Thermogalvanic Seebeck Coefficient”, *S*_e_;
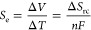
where Δ*S*_rc_ is the entropy difference between both redox states of the pair, *n* is the number of electrons transferred, and *F* is the Faraday constant.^7^ The *S*_e_ also could be affected by other contributions, such as the Soret effect, which can alter the potential difference.^5,6^ Therefore, the characterization and interpretation require careful attention. It is important to note that the power output (*P*_out_ = *V·I*) of a thermocell is dependent on both the voltage and the current that is permitted to flow through it. The current is limited by the total cells’ resistance, *R*_cell_ = *R*_et_ + *R*_el_, where *R*_et_ stands for electron-transfer resistance and *R*_el_ is for the electrolyte resistance.

The electrical current produced in a thermocell depends on different factors, including the coefficient itself, *S*_e_, the temperature difference, Δ*T*, and geometrical relation. Additionally, the current also depends on the kinetic redox process (electron transfer at the electrode) and on the mass transport of redox pairs. The diffusion of pairs through the electrolyte determines the electrical output current. To search for the best performance of devices based on thermocells, it is essential to optimize the *S*_e_ coefficient, mass transport, and kinetics. In this sense, several strategies have been reported in the literature to enhance the *S*_e_ in liquid electrolytes. Many of these involve the use of typical redox pairs such as Fe^2+/3+^ or Fe(CN)_6_^3–/4–^. Some of these strategies alter the solvation shell of an aqueous electrolyte by incorporating different species such as urea, guanidium, inert ions, or organic solvents.^8−10^ Other strategies based on the phase separation phenomena, charge-additivity, or molecular recognition have also been recently reported in the literature.^4,11,12^

An alternative to commonly used liquid electrolytes, the gel-based electrolytes have recently emerged, providing outstanding advantages over the liquid-based electrolytes.^13−15^ These gel-based electrolytes can overcome drawbacks related to stability, management, and safety typically associated with conventional liquid electrolytes. Moreover, the ability to adjust their mechanical properties, such as softness, toughness, or strength, in a very broad range of values opens the possibility to design new wearable and flexible devices.^16^

It has been demonstrated that gelled systems are able to solvate high concentrations of electrolytes, comparable to the high concentrations achieved in aqueous thermocells.^17^ Additionally, gelled electrolytes hinder thermal transport and facilitate the retention of larger temperature gradients, which benefits the efficiency of the thermocells.^3,13,18^

While most studies have focused on the optimization of the *S*_e_ coefficient, which plays a major role in liquid-based thermocells, there are fewer studies that focus on the optimization of the current through the electrolyte as we analyze in detail in this study. This is important in all types of electrolytes, but it results critical in the gel-based ones. Recently, simultaneous thermogalvanic and thermocapacitive effects have been reported in cationic (anionic) hydrogels by utilizing cationic (anionic) redox pairs and carbon cloth electrodes.^3^ In this context, experimental studies that combine different techniques to provide fundamental knowledge about gel-based electrolytes are of great interest. Here, we have focused on designing hybrid hydrogels based on monomers with different functional groups with the aim of analyzing their suitability as soft electrolytes and optimizing their performance. Using platinum electrodes in the thermogalvanic characterization we perform, the electron transfer resistance (*R*_et_) is minimized. This simplification allows us to better understand the role played by the interactions between the redox pairs and the hydrogel, which is of fundamental importance. Through 3D-printing techniques, we perform a synthesis method that enables us to design precise and intricate models in a quick, reproducible, and straightforward manner. Moreover, this method also provides specific control of the final properties of the hydrogels, allowing us to elucidate the role played by the functional groups anchored in the matrix and the correlation with their thermogalvanic features. The assessment of mechanical properties confirms the reproducibility and stability of the synthesized thermogalvanic hydrogels. We have analyzed the role of water content (swelling degree), the redox pair concentration, and the polymer chain functional groups on the thermogalvanic Seebeck coefficient and the output power (*P* = *I·V*). We explored two distinct hydrogels and two different redox pairs. On the one hand, the hydrogel based on the [2-(acryloyloxy)ethyl]trimethylammonium chloride (AETA) monomer, referred as AETA hydrogels, contains positive charges (N^+^) chemically fixed at their branches through covalent bonds, thus forming a cation active network (CN). The chlorine counteranions are electrostatically associated with the nitrogen cations and are distributed in the electrolyte solution. This hydrogel is studied with the Fe^2+/3+^ and Fe(CN)_6_^3–/4–^ pairs. On the other hand, the hydrogel based on the vinyl diaminotriazine (VDT) group, named VDT hydrogels, is studied only with the Fe(CN)_6_^3–/4–^ pair due to the inconsistent results obtained with the Fe^2+/3+^ pair. By combining thermoelectrochemical and mechanical characterization with electron microscopy and Fourier-transform infrared (FTIR) experiments, we have observed the significant impact of charge interactions between cationic pairs and the electroactive cation network, which enhances the power output.

## 2 Materials and Methods

### 2.1 Preparation of Hydrogels

The synthesis of hydrogels employed in this study consists of a facile and inexpensive method that avoids the use of toxic solvents. This methodology has been formerly established by our research group in previous publications.^19,20^

#### 2.1.1 AETA-Hydrogels

**Figure 1:**
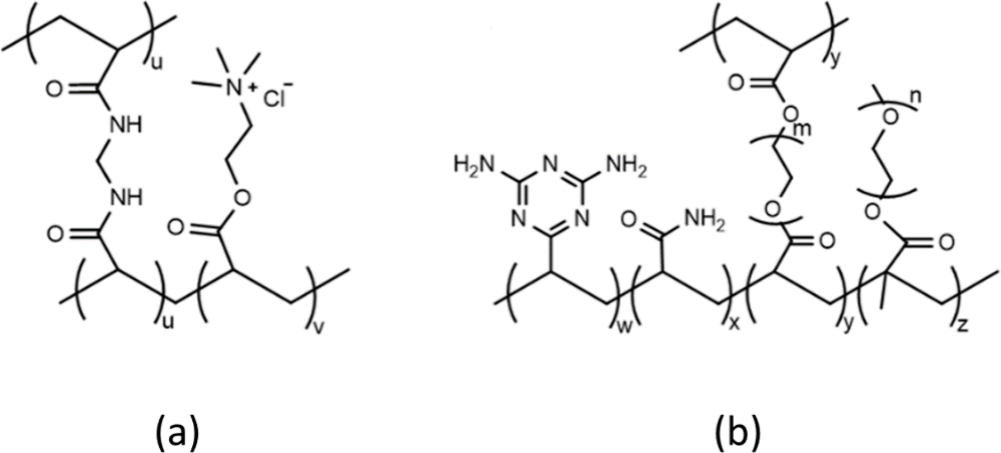
Representation of the chemical structures of (a) AETA and (b) VDT hydrogels.

The synthesis of the AETA hydrogels (Figure 1a) was carried out by sequentially adding and stirring the following compounds: 1280 mg of [2-(acryloyloxy)ethyl]trimethylammonium chloride (AETA) (80 wt % in water) (5.280 mmol), 2 mg of *N*,*N*′-methylenebis(acrylamide) (MBA) (0.012 mmol), and 4 mg of sodium 2,4,6-trimethylbenzoylphosphinate (NaTPO) (0.012 mmol) in 1 mL of Milli-Q water. The mixture was kept covered to prevent exposure to light before polymerization. Polymerization took place under an ultraviolet lamp in a silicone mold. After 1 min, hydrogels with the specific shape of the mold used for synthesis were obtained.

#### 2.1.2 VDT Hydrogels

The VDT hydrogel (Figure 1b) was synthesized by a 3D printer via in situ radical polymerization. The ink preparation involved stirring 150 mg (1.094 mmol) of VDT in 1 mL of dimethyl sulfoxide (DMSO) until the main monomer dissolved. Subsequently, the monomers were added in the following sequence: 135 mg (1.90 mmol) of acrylamide (AM), 70 mg (0.074 mmol) of poly(ethylene glycol) methyl ether methacrylate (PEGMA), 5 mg (0.007 mmol) of cross-linker polyethylene glycol diacrylate (PEGDA), and 3.03 mg (0.008 mmol) of the initiator lithium phenyl-2,4,6-trimethylbenzoylphosphinate (LiTPO). This monomer mixture was then introduced into the vat and polymerized using the Photon S Anycubic printer with a 405 nm wavelength. The resulting organogels underwent a washing process by replacing Milli-Q water (600 mL) twice a day for at least 4 days to remove DMSO and any unreacted monomers.

The electrolyte incorporation into the gels was done by drying the hydrogels in an oven (35 °C) until their mass no longer changed (xerogel) during at least 24 h. Then, the xerogel is introduced into a precise solution volume of Fe(II) and Fe(III) for either FeCl_2/3_ or K_3/4_[Fe(CN)_6_] for the AETA and VDT hydrogels during 48 h to achieve simultaneously the swelling degree and the molar concentration. This procedure ensures the full incorporation of the solution into the gel, thereby avoiding any alteration in the concentration.

The optimal printing parameters for VDT hydrogels were: Layer Thickness: 0.1 mm; Normal Exposure Time: 60 s; Off Time: 1 s; Bottom Exposure Time: 80 s; Bottom layer: 6. Z Lift Distance: 6 mm; Z Lift Speed, and Z Refract Speed: 6 mm/s.

### 2.2 Structural Characterization

Samples were analyzed by field emission scanning electron microscopy (FE-SEM) with a *Zeiss GeminiSEM* 500 system equipped with *cryo-SEM QUORUM* for low temperature and operating in the In-lens detector mode with an accelerating voltage of 2 kV. For the FE-SEM analysis, samples were prepared by the standard lyophilization process, which removes the water by sublimating it in the freeze samples and thus preserving the microstructure of the organic support. To estimate the pore size distribution, we measured the pore sizes of a significant number of hydrogel samples and created a histogram of the frequency counts against bin centers. We then fitted a Gaussian curve to the data. The mean pore size was considered the center of the distribution with the associated error given as the standard deviation of the fit.

FTIR spectroscopy was also performed on lyophilized and frozen samples by an IRAffinity-1S in transmission mode between 500 and 4000 cm^–1^.

### 2.3 Mechanical Characterization

The testing machine *MecmesinTM* Multitest 2.5-I, with a load cell rated for 50 N, was used to measure forces and displacements in the hydrogels. Cylindrical hydrogel samples of size 5 mm in height and 10 mm in diameter were compressed between two plates. These hydrogel samples were uniaxially compressed between two plates at a rate of 6 mm/min until reaching 40% strain. Young’s modulus was calculated from the linear region of these curves, corresponding to deformations ranging from 2 to 10%. Then, fatigue tests were conducted, involving multiple compression cycles performed at a speed of 30 mm/min. Each cycle encompasses compression from 0 to 40% displacement followed by relaxation from 40 to 0% displacement. The cylindrical samples measure 5 mm in height and 10 mm in diameter. Fatigue tests were conducted to perform 100 compression cycles on each hydrogel.

### 2.4 Thermogalvanic Characterization

The open circuit thermovoltage (*V*_op_) was obtained directly by measuring the voltage difference with a Keithley 6220 nanovoltimeter across the hydrogel with two platinum electrodes. The temperature difference was applied and controlled by two Peltier cells acting as cold and warm sources in contact with the Pt electrodes. The power density was characterized by measuring current vs voltage curves by applying Δ*T*, which offers both the short circuit and the open voltage features with a Keithley 2400 source meter with the thermal setup described above. During the measurements, the evolution on time was monitored; therefore, current vs voltage measurements were taken every 3 s during long time delays (above 10 min) to obtain the true stationary current through the hydrogel.

## 3 Results and Discussion

Both the AETA- and VDT-based hydrogels provide a stable and soft framework that is able to dissolve the different ions participating in the confined water during the thermogalvanic process. Despite the excellent mechanical properties of these both types of hydrogels, which have been previously analyzed by our group,^19,20^ the incorporation of the redox pair may engage the ability to retain water in the hydrogels, as it will be discussed. A significant difference in the water content would modify the current output, invalidating any power comparison. Thus, prior to the thermogalvanic characterization, the effect of the redox pair on the water content together with their mechanical features has been carefully examined. The water content is evaluated by means of the swelling degree, , where *W*_s_ and *W*_0_ refer to the weight of the swelled and dry hydrogel, respectively. Table 1 contains the relevant information related to the concentration of the redox pair and the swelling degree of each sample analyzed in this study. Here, it is important to distinguish between those cases where the maximum swelling degree has been reached (marked with ‘a’ next to the SD value) from those cases in which the swelling degree is adjusted below its maximum to ensure similar amounts of water in all samples.

**Table 1:** Redox Pair, Molar Concentration Swelling Degree, and Mean Pore Size of Samples Analyzed in This Study^a^

sample	redox pair	molar concentration	swelling degree	mean pore size (μm)
AETA_Fe_5	Fe^2+/3+^	0.05	5	26 ± 4
AETA_Fe_15	Fe^2+/3+^	0.15	5	52 ± 4
AETA_FeCN_5	Fe(CN) ^3–/4–^	0.05	5	20 ± 1
AETA_FeCN_15	Fe(CN) ^3–/4–^	0.15	3.2^a^	2.25 ± 0.04
VDT_FeCN_5	Fe(CN) ^3–/4–^	0.05	3.3^a^	15.9 ± 0.4
VDT_FeCN_15	Fe(CN) ^3–/4–^	0.15	2^a^	--

a Maximum swelling allowed.

In the AETA hydrogels containing Fe^2+/3+^, the SD was adjusted to 5 for both concentrations, and no effect on the ability of the gel to retain water was found. In the AETA hydrogels with the ferri/ferrocyanide pair, the swelling degree can be adjusted to 5 for the lowest concentration, whereas for the higher concentration, the maximum SD is lower than this value. Therefore, an interaction between the pairs and the gel is inferred from this result. The last occurs for the VDT hydrogels containing the ferri/ferrocyanide pair, where it is observed that the higher the redox pair concentration, the lower the maximum swelling degree.

**Figure 2:**
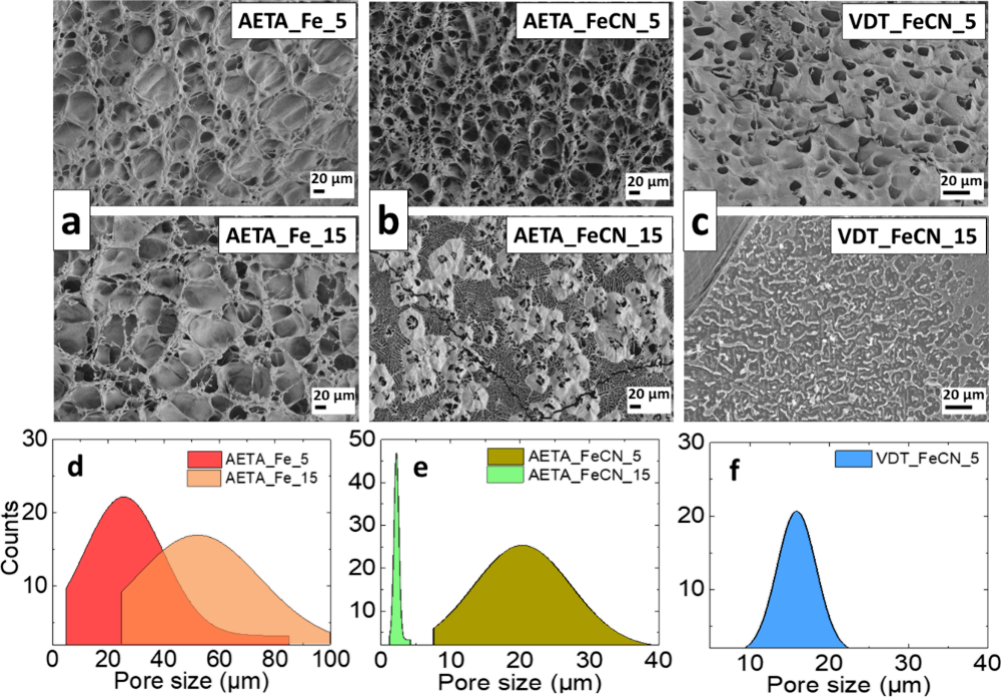
SEM images for the samples analyzed in this study (a) AETA_Fe, (b) AETA_FeCN, and (c) VDT_FeCN. The upper row corresponds to hydrogels with a 0.05 M pair concentration, and the middle row corresponds to hydrogels with a 0.15 M concentration. The lower row (figures d–f) displays the pore size distribution of the samples on top.

**Figure 3:**
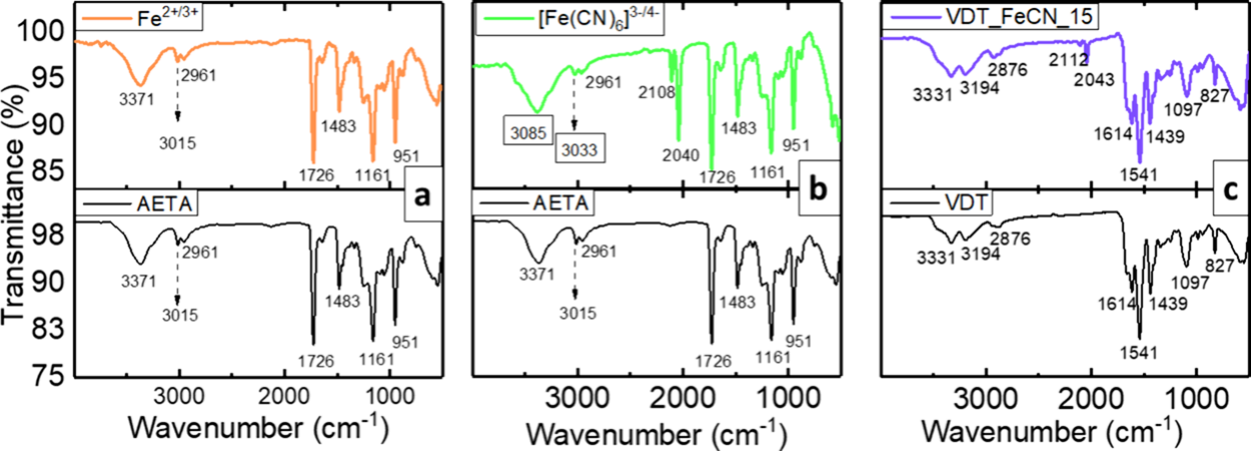
FTIR transmittance spectra for (a) AETA_Fe_15, (b) AETA_FeCN_15, and (c) VDT_FeCN_15 hydrogels in the upper row compared with the native hydrogels in the lower row.

The correlation between the maximum SD and the redox pair concentration could have a different source depending on each hydrogel and pair. To address this issue, scanning electron microscopy (SEM) images were examined to inspect the microstructure of the electrolyte hydrogels that were analyzed here. In Figure 2, the SEM images obtained for AETA and VDT hydrogels containing the analyzed concentrations (0.05 and 0.15 M) for each corresponding pair redox are compared. As hydrogels consist of a 3D polymer network that is flexible and soft, their microstructure varies depending on the containing monomer and pair. This fact is evident by comparing the mean pore sizes summarized in Table 1. In the AETA and VDT, containing the ferri/ferrocyanide pair (Figure 2b,c, respectively), the mean pore size reduces as the pair concentration enhances. However, the AETA hydrogel containing the Fe^2+/3+^ (Figure 2a) displays the opposite trend; i.e., the pore size increases as concentration increases. In this case, the enhancement of the pore size is noticeable, leading to increased water retention ability in the gel with higher redox pair concentrations. The overall trend can also be compared through the pore size distribution in Figure 2d–f. This feature will be critical for its thermoelectrochemical behavior, as we discuss next. Aiming to elucidate the source of both opposite trends with regard to the mean pore size and the presence of pairs, an FTIR analysis was performed on these samples. In Figure 3, the transmittance of the peaks corresponding to the AETA hydrogel with the Fe^2+/3+^ (Figure 3a) and the Fe(CN)_6_^3–/4–^ (Figure 3b) pairs, as well as those peaks corresponding to the VDT hydrogel with Fe(CN)_6_^3–/4–^ (Figure 3c), are compared to their native hydrogels. The FTIR spectra do not show differences between the native VDT hydrogel and the one containing Fe(CN)_6_^3–/4–^ (Figure 3c), indicating an absence of any discernible interaction between the pair anions and the organic network.^21^ However, in the case of the AETA hydrogel, a clear displacement of the band corresponding to the quaternary nitrogen (3015 cm^–1^) is observed due to the presence of the Fe(CN)_6_^3–/4–^ pair (Figure 3b), which points toward the coordination between the positive nitrogen and the negative redox pair.^22^ Finally, the AETA hydrogel containing Fe^2+/3+^ (Figure 3a) does not show any displacement.

**Figure 4:**
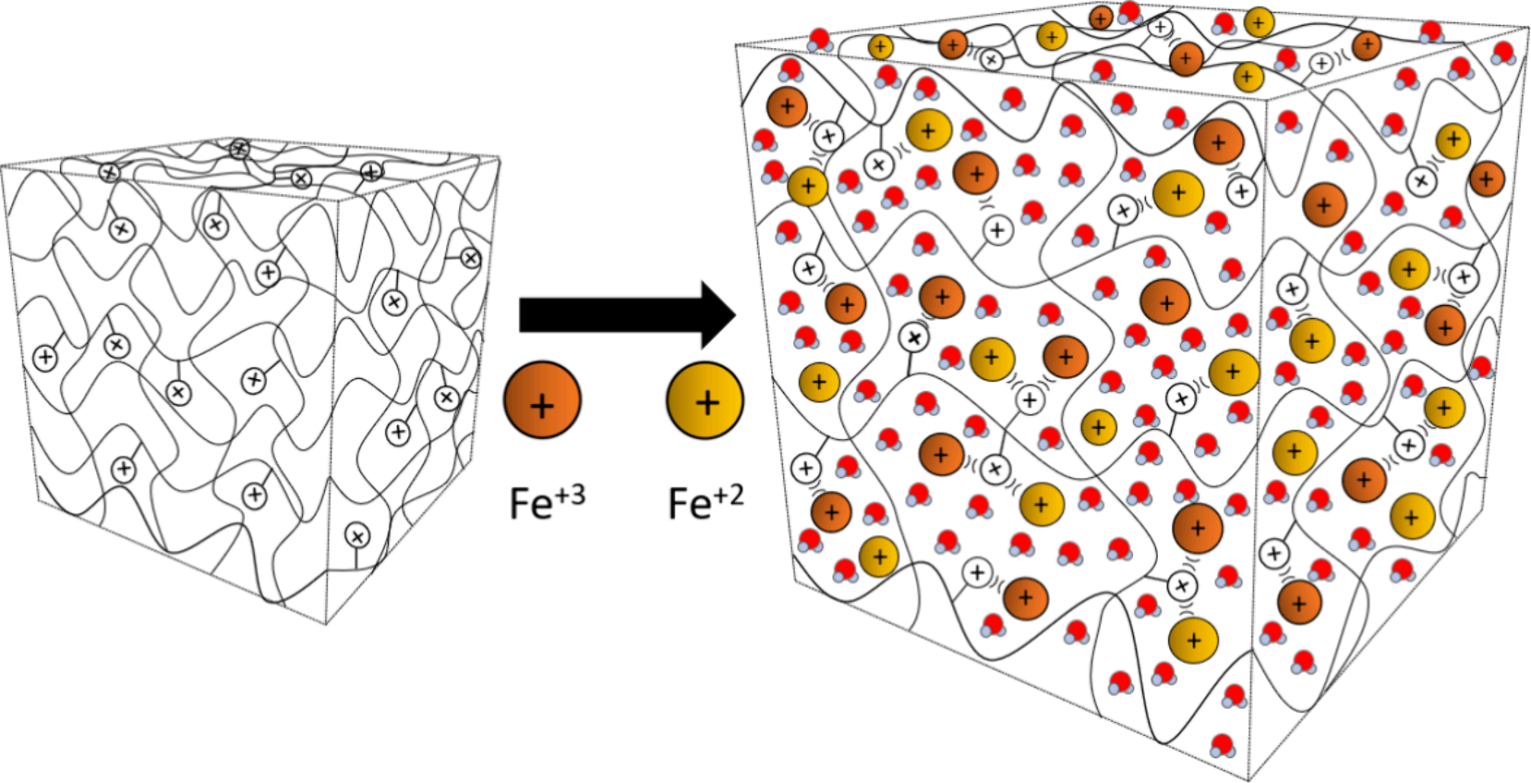
Schematic diagram of the AETA-Fe hydrogel where the electrical repulsive interaction between cationic branches (N^+^) and Fe^2+/3+^ is indicated.

In short, the FTIR spectra demonstrate that there is a clear interaction just for the Fe(CN)_6_^3–/4–^ complex in the AETA-based hydrogel, which is a cation network. Recent studies in the literature report on the specific ion effects in electrolytes and the role played by salts on the swelling degree.^23−25^ Thus, in the AETA_FeCN hydrogels, the attractive interaction between the negative charges of the Fe(CN)_6_^3–/4–^ and the positive charges (N^+^) on the AETA branches reduces the pore size and reduces its maximum swelling degree. The hydrogels formulated with VDT, whose network is neutral, do not interact with the pairs, and they exhibit lower swelling due to the higher ionic strength resulting from their increased concentration, as observed in similar compounds.^3^ On the other hand, in the AETA_Fe hydrogels, the repulsive interaction between the cation branches and the Fe^2+/3+^pairs causes the pore to increase, which in turn must raise the maximum swelling degree above the adjusted SD. The larger mean pore size facilitates the diffusion of the pairs;^26^ moreover, the repulsive interaction between the matrix and the pairs might also contribute to this diffusion. A simplified scheme of the cationic thermogalvanic (TG) hydrogel, showing the repulsive interactions between the Fe^2+/3+^ pairs and the positive branches in the AETA hydrogel, is shown in Figure 4.

**Figure 5:**
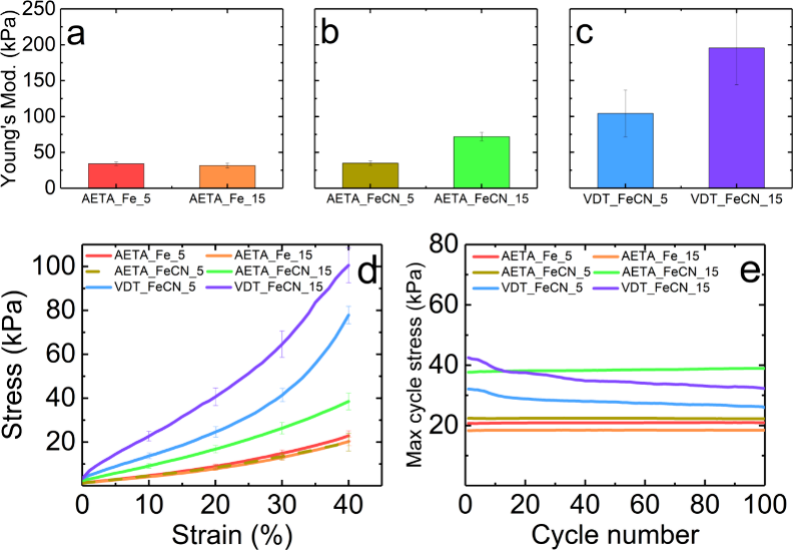
Mechanical properties of the AETA and VDT hydrogels. Young’s modulus of (a) AETA_Fe, (b) AETA_FeCN, and (c) VDT_FeCN. (d) Stress–strain curves of both hydrogels with different concentrations of redox pair. (e) Cyclic fatigue test for both hydrogels with different concentrations of redox pair.

To evaluate the mechanical properties of the synthesized hydrogels, compression tests were performed to probe their elasticity; see Figure 5. Stress–strain curves, shown in Figure 5d, were recorded during experiments conducted at room temperature, accounting for different degrees of swelling and concentrations of the redox pair. The diverse redox pairs and concentrations not only influence the thermogalvanic effect but also serve as cross-linkers within our systems. For the AETA hydrogel, the Young’s modulus with the Fe^2+/3+^ redox pair was 34.2 kPa at a concentration of 0.05 M and 31.6 kPa at a concentration of 0.15 M (Figure 5a). When moving to the Fe(CN)_6_^3–/4–^ redox pair, the Young’s modulus was 34.9 kPa for 0.05 M and 71.9 kPa for 0.15 M (Figure 5b). This increase also arises from the interaction between the negative ions and the positive charges of the hydrogel, leading to a reduction in the maximum swelling degree at this concentration and redox pair while rendering the gel stiffer. On the other hand, VDT hydrogels with the Fe(CN)_6_^3–/4–^ redox pair exhibit Young’s modulus values of 104.1 kPa at 0.05 M and 195.5 kPa at 0.15 M (Figure 5c). These results demonstrate that AETA hydrogels have lower Young’s modulus, making them more flexible compared to VDT hydrogels. Conversely, VDT hydrogels exhibit higher Young’s modulus values, indicating greater rigidity and less deformability, enabling them to resist higher loads before deformation occurs. It is also essential to investigate how materials behave under repeated deformations, depending on their intended use in devices. Thus, fatigue tests were conducted to undergo 100 compression cycles on each hydrogel. As depicted in Figure 5e, AETA hydrogels with varying concentrations and redox pairs exhibit no significant loss in their maximum stress over successive deformation cycles. This observation suggests efficient dissipation, rendering the hydrogel durable even when compressed up to 40% of its initial height as the hydrogel returns to its initial state. In contrast, VDT hydrogels with Fe^3+/2+^ exhibit a slight decrease in their maximum stress. Specifically, the strength decreases from 31.9 to 26.3 kPa for the 0.05 M concentration (17% decrease from its initial value) and from 41.9 to 32.7 kPa for the 0.15 M concentration (22% decrease from its initial value).

**Figure 6:**
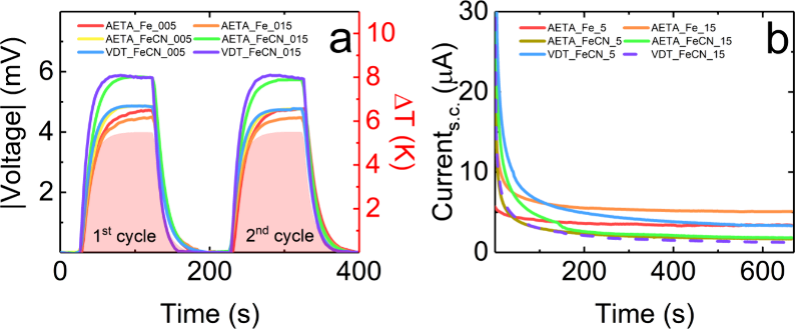
(a) Absolute voltage differences (left axis) when a temperature gradient is applied (right axis) between electrodes as a function of time. The solid pink area represents the switch on/off for each cycle. (b) Short-circuit current as a function of time for an applied temperature difference of 7 K. Both plots refer to all the samples analyzed in this study.

To perform the thermogalvanic characterization of these hydrogels, they were placed between two Pt electrodes contacted with two Peltier stages, allowing the application of the temperature gradient. The voltage drop in a constantan wire across independent Pt electrodes in contact with the Peltier devices provides the temperature difference between both ends of the hydrogel. Figure 6a shows the comparison of the absolute voltage difference when a temperature difference is applied over two consecutive cycles, indicating the reproducibility and stability of the measurements across all the samples analyzed. Additionally, Figure 6b displays the short-circuit current measured as a function of time for an applied temperature difference of 7 K.

**Figure 7:**
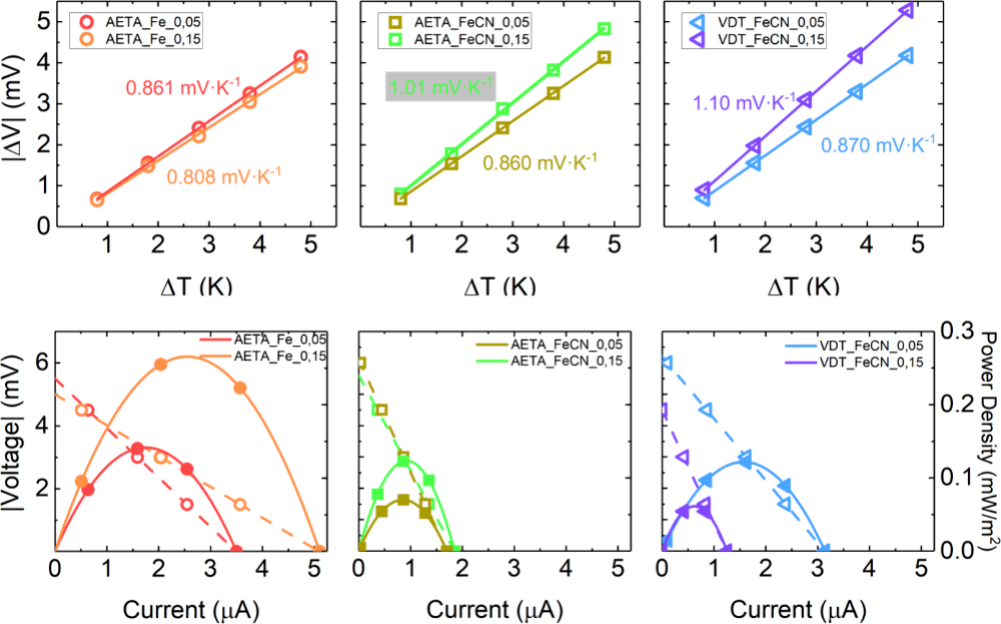
Upper panel: thermogalvanic output voltage (*V*_op_) as a function of the temperature difference for the samples studied in this article. Lines represent linear fits to the experimental points. Lower panel: voltage output and corresponding power density obtained for a temperature difference of 7 K.

The output voltage (absolute value) for various temperature differences ranging from 1 to 5 K is shown in Figure 7 (upper panel). The slope extracted from these plots provides the thermogalvanic Seebeck coefficient (*S*_*e*_). There are no significant differences between the measured *S*_e_ coefficients of the samples studied. However, there is a slight trend for both hydrogels containing the Fe(CN)_6_^3–/4–^ pair, in which higher concentrations produce higher *S*_e_ coefficients (between −0.86 and −1.1 mV·K^–1^). Besides, the AETA hydrogel containing Fe^3+/2+^ shows almost the same values of *S*_e_ for both concentrations analyzed.

Finally, the power density was evaluated by measuring the corresponding current–voltage curves under a constant temperature difference of 7 K. Figure 7 (bottom panel) shows the cell voltage as a function of the current and the corresponding power density (mW/m^2^). From these plots, it is observed that maximum power densities are obtained for the two AETA hydrogels containing the Fe^3+/2+^ pair. In which it is satisfied that a larger pair concentration produces the maximum power density output, being 0.27 and 0.14 mW/m^2^ for the 0.15 and 0.05 M concentrations, respectively. The AETA hydrogels containing the Fe(CN)_6_^3–/4–^ pair follow this trend, i.e., the larger concentration produces larger power but with lower overall values, 0.12 mW/m^2^ for 0.15 M and 0.07 mW/m^2^ for 0.05 M, which represents roughly 50% of the power outputs for the AETA_Fe compounds. Finally, the VDT_FeCN hydrogels display the opposite trend, and a higher power output is obtained for the sample with a lower concentration. In Table 2, the results obtained for the *S*_e_ and the maximum power outputs are summarized. These results point out that the main factor that determines the power output in these cases is the current instead of the *S*_e_. As the current is limited by the ability to retain water, the pore size, and the diffusion of the pair, this would explain why those hydrogels containing the Fe(CN)_6_^3–/4–^ pair display lower output values. In the case of the VDT hydrogel, this effect is dramatic, causing the values to plummet. In contrast, due to the cation network effect with the cationic Fe^2+/3+^ pair, diffusion is favored in this case, resulting in a beneficial impact on power output. Considering that the current results from the thermogalvanic effect at the electrodes, the counterions dissolved in the electrolyte just balance the charge to maintain neutrality and do not participate in the power output.

**Table 2:** Values of Thermovoltage (*S*_e_) and Maximum Power Output Obtained for the Samples Studied

sample	open circuit thermovoltage (mV/K)	max. power output (mW/m^2^)
AETA_Fe_5	0.861 ± 0.06	0.14 ± 0.02
AETA_Fe_15	0.808 ± 0.05	0.27 ± 0.03
AETA_FeCN_5	0.860 ± 0.05	0.07 ± 0.01
AETA_FeCN_15	1.01 ± 0.08	0.12 ± 0.02
VDT_FeCN_5	0.870 ± 0.05	0.12 ± 0.02
VDT_FeCN_15	1.1 ± 0.09	0.06 ± 0.01

The power output obtained for the hydrogels analyzed here is moderate but, considering the lower concentrations utilized, they are in the range of similar previously reported results.^3,27,28^ The interest of the present study should be in the effect of the use of cationic hydrogels containing cationic redox pairs on the current and, consequently, on the potential output. Future experimental studies combining this approach with different strategies will be of great interest for the realization of suitable and competitive devices based on thermogalvanic gelled electrolytes.

## 4 Conclusions

In conclusion, we have developed hydrogels containing different functional groups through a facile and clean synthesis process, and we have explored their ability to retain different concentrations of electrolytes. The analysis of the microstructure through SEM images shows a clear relation between the mean pore size and the sign of the redox pair for the cationic hydrogel (AETA hydrogel). Thus, in the presence of the cationic pair, the increase in the size of the pores facilitates the ability to contain larger amounts of water and, in consequence, favors the ion mobility. The FTIR spectra show an interaction between the anionic Fe(CN)^3–/4–^ pair and the cationic hydrogel which originates the pore size reduction. It is also reported that the sign of the redox pair influences the mechanical properties of the hydrogels studied. Finally, from the thermogalvanic characterization, which includes current–voltage curves, an increase in power output is observed due to an upturn of the current for the cationic hydrogel containing cationic pairs. Therefore, the strong influence of the sign of the cation on the power density is evident in the case of the AETA hydrogel. The mechanism proposed accounts for the repulsion between the positive branches of the polymer and the cationic Fe^2+/3+^ pair, facilitating conduction and consequently improving power densities.
